# Hitting the nail on the head: combining oncolytic adenovirus-mediated virotherapy and immunomodulation for the treatment of glioma

**DOI:** 10.18632/oncotarget.20810

**Published:** 2017-09-11

**Authors:** Wojciech K. Panek, J. Robert Kane, Jacob S. Young, Aida Rashidi, Julius W. Kim, Deepak Kanojia, Maciej S. Lesniak

**Affiliations:** ^1^ Department of Neurological Surgery, Northwestern University, Chicago, IL, 60611, USA; ^2^ Pritzker School of Medicine, University of Chicago, Chicago, IL, 60637, USA

**Keywords:** combinatory therapy, glioma, immunomodulator(s), checkpoint blockade, oncolytic adenovirus

## Abstract

Glioblastoma is a highly aggressive malignant brain tumor with a poor prognosis and the median survival 14.6 months. Immunomodulatory proteins and oncolytic viruses represent two treatment approaches that have recently been developed for patients with glioblastoma that could extend patient survival and result in better treatment outcomes for patients with this disease. Together, these approaches could potentially augment the treatment efficacy and strength of these anti-tumor therapies. In addition to oncolytic activities, this combinatory approach introduces immunomodulation locally only where cancerous cells are present. This thereby results in the change of the tumor microenvironment from immune-suppressive to immune-vulnerable via activation of cytotoxic T cells or through the removal of glioma cells immune-suppressive capability. This review discusses the strengths and weaknesses of adenoviral oncolytic therapy, and highlights the genetic modifications that result in more effective and targeted viral agents. Additionally, the mechanism of action of immune-activating agents is described and the results of previous clinical trials utilizing these treatments in other solid tumors are reviewed. The feasibility, synergy, and limitations for treatments that combine these two approaches are outlined and areas for which more work is needed are considered.

## INTRODUCTION

Glioblastoma is a highly malignant brain tumor with an extremely dismal prognosis. Standard methods of treatment, which often involve surgical resection, chemotherapy, and radiotherapy, prolong patient survival but only to an extent. With this, survival is often limited to a little over one year following diagnosis [1, 2]. Because of the aggressive nature of this disease and the limited treatment options available, new and further developed anti-tumor modalities of care are desperately needed. One difficulty, notwithstanding all other considerations, related to the development of novel treatment modalities is the unique immune repertoire and tumor microenvironment of glioma [3–5]. As a vastly heterogeneous tumor, glioblastoma (previously referred to with the descriptor of ‘multiforme’ for this very reason) has a tumor microenvironment that is immunosuppressive such that a conventional immune response against the tumor does not ordinarily take place [2–4]. Standard modes of treatment do not modulate the tumor microenvironment in this regard. Fortunately, progressively developed modes of combating these hurdles have come to the forefront such as to enable the active targeting of the tumor as well as its immunosuppressive microenvironment. Oncolytic virotherapy has been developed as a therapeutic modality that utilizes viral infectivity to specifically target the tumor [6–10]. The tropism of the virus can be modified in order to enhance the viral infectivity of neoplastic cells specifically while simultaneously limiting the infection of non-neoplastic tissue. This specific oncolytic activity proves to be a gainful approach for attaining a marked anti-tumor response [9, 11–16]. Clinical trials of such studies as these have demonstrated some success that further supports the advance of these therapies while also encouraging the discovery of other experimental developments that may increase their efficacy [17–22] .

As noted already, the uniquely distinct tumor microenvironment of glioma creates immunosuppressive conditions that ordinarily do not allow for significant anti-tumor immune responsiveness. Immunotherapy strives to overcome this intrinsic tumor immunosuppression and promotes a defined immune response against the tumor through immune system modulations [23–25]. Defined by the presence of regulatory T cells, M2 macrophages, and myeloid-derived suppressor cells (MDSCs), the immunosuppressive glioma microenvironment may prove susceptible to approaches that promote an anti-tumor immune response through T cell activation [23–25]. Immunomodulation of the expression of cell surface receptors such as CTLA4, PD-1, and 4-1BB may be capitalized on to thus yield a T cell immune response in order to achieve an anti-tumor response [26–29]. Several clinical trials have utilized this mode of a novel therapeutic modality, some of which have garnered appreciable success [30–38].

Together, oncolytic virotherapy and immunotherapy have proven to be viable targeting options for the treatment of glioma. Their combination represents a promising treatment modality that has the potential for success in the clinic in ways that have not before been described. This could result in the synergistic destruction of the tumor, thus augmenting the survival benefit for patients, while simultaneously minimizing the risk of systemic side effects and toxicities. Ongoing and upcoming clinical trials will further explore their clinical benefit while studies attempting to augment their efficacy and demonstrate their applicability for the treatment of a disease where new treatment options are desperately needed.

### Oncolytic Adenoviral Virotherapy in Glioma

Human adenovirus serotype 5 (Ad5), is one of the most common virus types used in glioma therapy since the biology of this double-stranded DNA virus is well studied and its use has been widely applied for gene therapy approaches [10, 15, 39]. Moreover, Ad5 is comparably easier to genetically modify without affecting its structural stability and host cell infectivity, which allows for redirecting its viral tropism (cell-specific targeting) and for restricting its replication (cell-specific lysis) [40–42]. Due to its well studied unique characteristics, proven safety, and efficacy found during the past few decades, many cancer-specific, targeted oncolytic adenoviruses are currently being tested in clinical trials [17–22, 43–45].

### Types of oncolytic adenoviral vehicles

Different modifications of Ad5 tropism have brought current research studies a step closer to a well designed, targeted oncolytic therapy against glioma. Selective tropism, the ability to bind to and enter target cells, is the ultimate goal of effective viral infection for therapeutic purposes [9, 12, 13, 19, 40, 46]. The selective tropism of adenovirus is dictated by the fiber-knob domain [47]. In this context, several changes in this structure have been proposed to achieve targeted interaction in glioma cells. Since most cancerous cells, including much of those involved in glioma, lack the primary adenovirus receptor, CAR (*Coxsackievirus and adenovirus receptor*), various viral modification approaches are required and thus have been made to achieve efficient infection [11, 48]. Given the presence of surface proteins overexpressed primarily on the surface of glioma cells, it is possible to develop targeted viral vectors by incorporating a binding moiety of these surface proteins onto the adenoviral fiber for both gene therapy and oncolytic virotherapy [49, 50].

**Figure 1:**
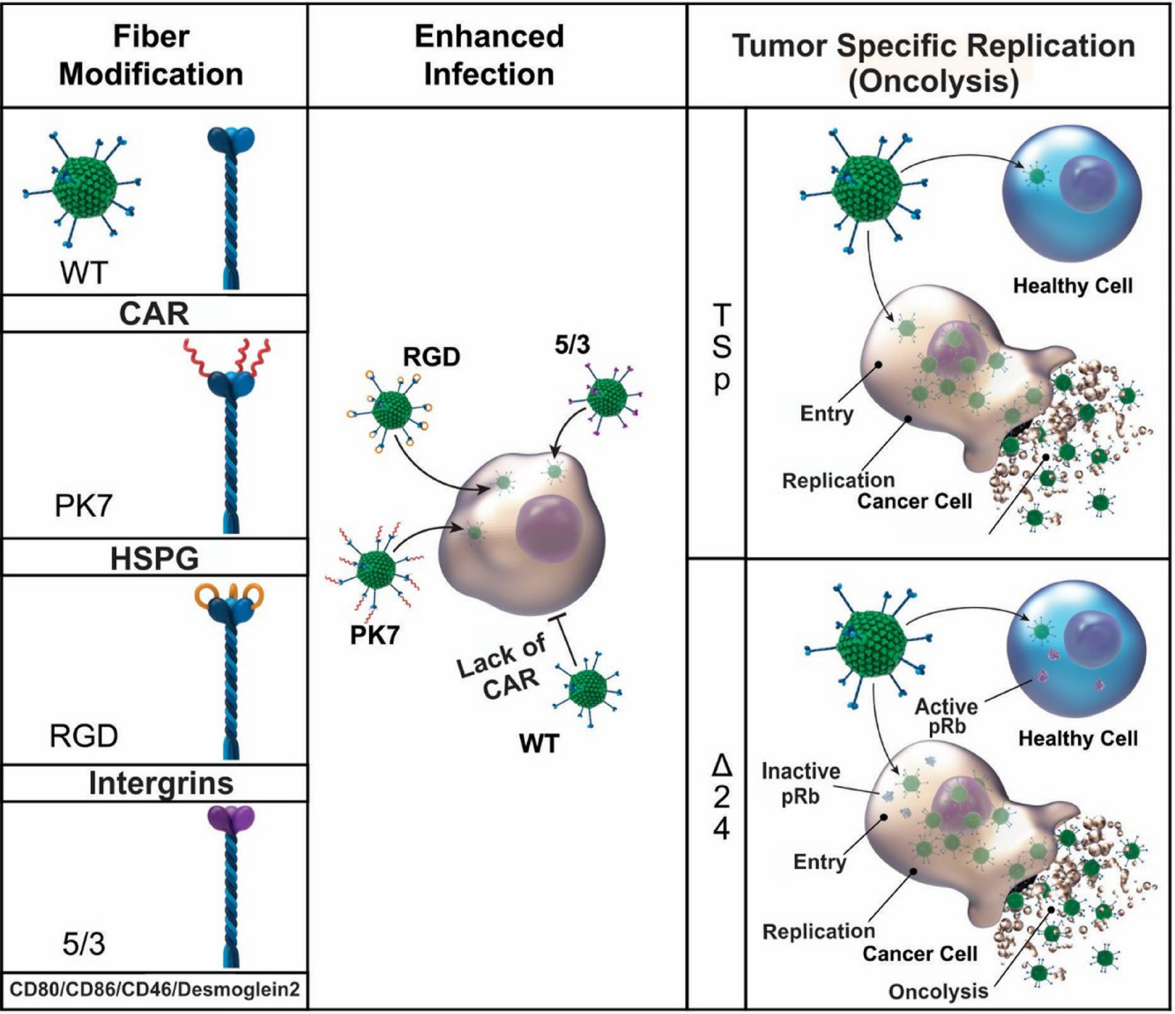
A schematic diagram of infectivity-enhanced fiber modifications and tumor-specific replication of adenovirus Various viral modification approaches have been constructed to achieve efficient infection and cancer-specific replication. Among the various types of viral modification, the most common approaches for tropism modification (pk7, RGD, and 5/3) and tumor-restricted replication (tumor-specific promoter and delta24) are depicted.

Among them, Ad5-pK7, Ad5.RGD, and Ad5/3 represent some of the leading contenders which bind to anionic surface proteins, integrins, and CD80/CD86/ [51, 52]/Desmoglein 2 [53], respectively (Figure 1). These modifications have become important to achieve a high efficacy of transduction to glioma cells. Ad5-pK7 is an insertional modification, which has been created by incorporating seven poly-lysine (pK7) residues onto the fiber-knob domain [50, 54–56]. These residues (pK7) were found to be a motif that binds to over-expressed heparan sulfate proteoglycans (HSPGs) on the surface of glioma cells [57]. Because of the high level of binding affinity to these receptors, this configuration is known to be highly effective and, based on currently available research studies, has the highest infectivity among the tested adenoviral modifications (an average of 70% infectivity in tested glioma cell lines such as U87, U251, and patient derived GBM43) [17, 50, 54–56]. Based on prior work in this area, this Ad5-pK7-based approach is being used in a phase I clinical trial for patients with glioma [17].

Another approach involves targeting integrins, which are overexpressed on many cells, including glioma cells. Specifically, the integrins αvβ3 and αvb5 have been associated with enhanced tumor angiogenesis and are therefore viable targets for oncolytic virotherapy [58, 59]. Moreover, integrins αvβ3 and αvb5 were found to be over-expressed in glioma cells. Because of this, adenoviral vector Ad5.RGD was constructed by inserting an arginine (R)-glycine (G)-aspartic (D) acid-4C complex at the level of HI-loop in the Ad5 fiber-knob domain. This modification has led to the enhanced infection of cancers that overexpress this set of integrins [39, 46]. Many clinical trials including glioma, ovarian cancer, and other selected gynecologic cancers are being treated using this enhanced infectivity motif incorporated through Ad5RGD viral vehicles [60–66].

A third approach involves a chimeric fiber protein construct termed Ad5/3 [14, 39, 67, 68]. In this particular scenario, the fiber knob domain of Ad5 has been replaced with that of Ad3, allowing for enhanced transduction of cells overexpressing CD 80, CD86, and Desmoglein 2 [49, 50, 52]. Binding activity and replication efficiency of these chimeric constructs is significant, and a relevant oncolytic effect has been observed in murine cancer models, including glioma, pancreatic, ovarian, and prostate cancers [69–73].

All mentioned modifications at the level of the Ad5 fiber domain have become means of achieving more effective infection of glioma cells. By combining the available fiber modifications with specific promoters to drive the expression of replication of essential genes which will be discussed in later section, many have become excellent tools with remarkable oncolytic capabilities for glioma therapies [6–8, 11, 12, 16, 19, 40–42, 46, 49].

### Restricted replication, cancer specific oncolysis of Adenovirus

Firstly, an adenovirus releases its progeny through the lysis of the host cell rather than exocytosis. As such, proper control of viral replication restricts host cell lysis to cancerous cells. To achieve tumor-restricted replication, many genetic modification approaches have been applied to the adenoviral vector based on the unique biology of neoplastic cells such as hyperactive promoter/mutations of gene(s) [39, 46, 74, 75] (Figure 1).

One most common approaches is to regulate the expression of adenoviral replication essential gene sequence, called early gene 1 (E1) under a tumor-specific promoter such as Survivin, CXCR4, or midkine. These tumor-specific promoters are known to be hyperactive in tumor cells (‘tumor always on’ status) while having nominal activity in normal cells. For instance, Survivin, a member of the inhibitor of apoptosis (IAP) family, has been shown to have elevated expression in glioma with a correlated association of poorer prognosis and increased rates of recurrence [39, 46, 74, 75]. Therefore, by taking advantage of this tumor-specific transcriptional regulation, replication-mediated oncolysis can be restricted only to cancerous cells.

Alternatively, adenoviral replications can be restricted to neoplastic cells by taking advantage of the malfunctioning cellular transcriptional machinery: inactivation of retinoblastoma protein (pRb) and p53 in cancer cells [76–78]. Upon the entry into normal cells, the first protein expressed from adenoviral DNA is the early gene 1 (E1) sequence, which produces E1A and E1B proteins that, among other things, bind to pRb and p53, respectively. This interaction continuously turns on cellular transcriptional machinery and allows for the massive production of viral proteins for the generation of its progeny. However, when a deletion of the binding sites for these proteins (24 nucleotides (Δ24) on E1A) or deletion of E1B (dl1152) is made to the adenoviral essential gene sequence, subsequent viral protein production cannot take place unless the function of pRb and p53 have already been impaired [79–82]. Since the majority of neoplastic cells have non-functioning pRb and p53, the cancer-restricted replication of an adenovirus is achieved by utilizing this understanding of the natural interaction between an adenovirus and relevant cellular machinery [79–82].

Additionally, although it is not direct adenoviral modification mediated oncolysis, incorporation of cytotoxic gene(s) such as HSV- thymidine kinase (tk) into a cancer specific adenovirus, called Ad-TK have been used and shown its clinical efficacy in GBM [83–85]. The usages of adenovirus as gene therapeutic oncolytic approaches can be found elsewhere [86–89].

### Advantages and disadvantages of adenoviral vector usages for glioma therapies in a clinical setting

There are several well-established advantages of adenoviral vectors that make them ideal for clinical applications. First, they are considered to have no or low pathogenicity. Second, it is easy to produce clinically necessary high titers of viruses: an average 10^12^ viral particles per milliliter (vp/ml) on each purification when 10^9^∼10^10^ (vp/ml) is used in clinic. Third, the viral DNA is not integrated into the host chromosome such that a therapeutic gene of interest incorporated into the viral DNA is expressed only transiently. And most importantly, there is a high degree of customizability (flexibility for genetic modifications: structural modification and insertion of therapeutic genes–maximal insertion capacity of 7.5Kb) [6, 11, 13, 15, 19, 46, 49].

By taking advantage of these features, many adenoviral vectors have been investigated and demonstrated efficient cancer-specific therapeutic efficacy. Furthermore, this adenovirus-mediated oncolysis has been shown to induce a tumor-specific immune response through the release of tumor antigens [90–94]. Additionally, oncolytic therapy may sensitize cancerous cells in order to inhibit the DNA repair system upon conventional therapies such as chemotherapy and radiotherapy [95–97]. Given these promising findings, many clinical trials utilizing adenoviral agents for patients with gliomas are currently underway. A dose escalation trial (phase I) of ONYX-015 (oncolytic adenovirus with E1B mutation) showed no serious adverse effect upon the treatment (doses up to 10^10^ pfu) with 6.2 months of median survival [98]. Also, oncolytic adenovirus DNX-2401 (also known as Delta-24-RGD) based clinical trials are now actively recruiting subjects for phase I and II trials [61] (Table 1).

**Table 1:** Clinical trials of oncolytic adenovirus-based virotherapy in glioma

Trial number	Type of treatment	Phase	Therapeutic agent	Additional Information
NCT00805376	DNX2401 (Formerly Known as Delta-24-RGD-4C) + TMZ	Phase I	Ad.Delta-24-RGD-4C	Drug: DNX-2401
				Procedure: Tumor Removal
				* Completed
NCT02197169	DNX-2401 With Interferon Gamma (IFN-γ) for Recurrent Glioblastoma or Gliosarcoma Brain Tumors (TARGET-I)	Phase I	CRAd DNX-2401 (Formerly Named Delta-24-RGD)	Drug: Single intratumoral injection of DNX-2401
				Drug: Interferon-gamma
				* In the process of recruiting participants
NCT03072134	Neural Stem Cell Based Virotherapy of Newly Diagnosed Malignant Glioma	Phase I	Neural stem cells loaded with Ad5-pK7	Biological: Neural stem cells loaded with Ad5-pK7
				* In the process of recruiting participants
NCT02798406	Combination Adenovirus + Pembrolizumab to Trigger Immune Virus Effects	Phase II	CRAd DNX-2401	Biological: DNX-2401 and pembrolizumab
				* In the process of recruiting participants
NCT01301430	Parvovirus H-1 (ParvOryx) in Patients With Progressive Primary or Recurrent Glioblastoma Multiforme	Phase I/II	Parvovirus H-1	Drug: H-1PV
				* Completed
NCT01582516	Trial of a Conditionally Replication-competent Adenovirus (Delta-24-rgd) Administered by Convection Enhanced Delivery in Patients With Recurrent Glioblastoma	Phase I/II	Ad.Delta-24-RGD	Biological: delta-24-RGD adenovirus
				* Completed
NCT01956734	Virus DNX2401 and Temozolomide in Recurrent Glioblastoma (D24GBM)	Phase I	DNX-2401 (Formerly Named Delta-24-RGD)	Procedure: DNX2401 and Temozolomide
				* Ongoing trial

However, there are still some challenges facing adenoviral oncolytic therapy. For instance, the majority of the human population has been pre-exposed to various adenovirus serotypes and it has been shown that therapeutic efficacy of adenovirus based agents could be negatively affected by any pre-existing immunity [99, 100]. Also, due to the nature of adenoviral tropism, this viral vector can be sequestered in liver, causing hepatotoxicity when delivered systemically [101–104]. As a result, many efforts strive to overcome these problems in order to increase infection efficacy and limit biotoxicity [101–104] (ref). For example, modifying the adenoviral capsid protein of the hexon to be less immunogenic and liver tropic can help circumvent unwanted targeting effects [101–104] . Furthermore, limited penetration of oncolytic adenoviruses throughout the depth of the solid tumor mass has been reported which is an inherent problem concerning oncolytic adenoviral therapy for glioma [86, 105]. In this case, it has been found that boosting the immune response against tumor antigen(s) released during oncolytic activity or activating cytotoxic T cells could augment the efficacy of adenoviral oncolytic activity [105–107].

### Immunotherapy in Glioma

Immunotherapy has become a rapidly progressing field in the treatment of glioma. Immunotherapeutic efficacy has been shown to be less toxic and more specific in comparison to the other current treatment modalities presently available (resection, chemotherapy, and radiation) [108]. In the context of glioma, immunotherapy has been largely based on glioma vaccination and immune system modulation, which can regulate/suppress the immunosuppressive glioma microenvironment that is defined by the presence of regulatory T cells, M2 macrophages, and myeloid-derived suppressor cells (MDSCs) [109].

### Pulsed DC-based therapies

One of the predominant immunotherapies for glioma that is already in clinical trials for patient treatment, is the pulsed dendritic cell (DC) vaccine (Supplementary Table 1). As DCs are the most effective antigen presenting cells (APCs) capable of eliciting a specific cell-mediated anti-tumor immune response, this approach is an attractive strategy to activate anti-tumor immunity for a variety of cancer types. However, due to the lack of *in vivo* DC-specific delivery vehicles, multi-step *ex vivo* strategies are more common. In this case, the patient’s autologous DCs are collected via plasmapheresis, stimulated *ex vivo*, and then pulsed with tumor specific antigen or whole tumor cell lysate. Finally, activated and pulsed DCs are systemically injected back into the patient in order to activate the T cell immune response [23, 109].

Initially, this DC-based tumor vaccine utilized a selected tumor-specific, antigen (TA)-based DC pulsing method. However, as glioma is highly heterogeneous and there being difficulty in identifying a glioma-specific antigen, this approach showed very limited efficacy [110]. Alternatively, it is presently more common to use whole tumor cell lysates pulsed with prepared DCs in the hope that these pulsed DCs will preferentially present tumor-specific antigen(s) not self-antigen(s) that would eventually cause serious autoimmune disease [111]. Therefore, to overcome these current limitations, single-step, *in vivo* DC delivery vehicles that can facilitate highly efficient multi-epitope TA loading for maximal anti-tumor immunity are needed to achieve efficacious glioma immunotherapy. As expected, side effects of these DC-based immunotherapies have been reported including transient fever, autoimmune vitiligo in melanoma patients, mild headache, and erythema in glioma patients [111].

Regarding the tumor immune response after DC vaccination in clinical trials NCT00612001 and NCT00068510, study authors found that decreased Regulatory T (Treg) cell and Natural Killer (NK) cell populations is correlated with increased survival for study patients [112]. In another study (NCT00846456), authors showed that when DC vaccines are loaded with immunosuppressive glioma stem cells (GSC) mRNA, they can recognize and activate CD8+ T cells and NK cells, consistent with their preclinical results [113]. A different study (NCT00323115) examined the percentage and number of the Tregs post-treatment in which there was an increase in percentage of Tregs but not in their absolute numbers. They also showed that there was an increase in CD8+ memory cells and naive B cells [114].

### Immunomodulators

#### Cytotoxic T Lymphocyte-associated Antigen 4 (CTLA-4)

One of the costimulatory signals for T cell activation involves the binding of B7-1 or B7-2 cofactors to their receptor, CD28 (positive regulator of T cell activation).With higher affinity, these cofactors (B7s) also bind to CTLA-4, a key negative regulator of T cell activation. These higher affinity-based interactions with CTLA-4 cause the inactivation and cell cycle arrest of T cells. Therefore, the balance between positive and negative regulation is essential to generate and maintain the T cell-based immune response [34, 36, 37]. Based on this understanding, anti-CTLA-4 (inhibition of the negative regulator) has been shown to induce tumor antigen-specific T cell immunity (adoptive immunity) and many clinical trials have been developed using this anti-CTLA-4 based T cell immunity induction (Supplementary Table 1).

This method has been used as both a monotherapy and in combination with vaccines, radiation, chemotherapy, and surgery, demonstrating highly synergistic efficacy [109]. In a recent phase III clinical trial, ipilimumab (commercial anti-CTLA antibody) monotherapy showed an overall response rate of 10.9%. Although significant tumor regression has been observed in many cancer treatment such as melanoma, prostate cancer, malignant mesothelioma, and lung cancer, autoimmune side effects (thyroiditis, colitis, and vitiligo) were observed in certain subsets of patients but all patients recovered after discontinuation of further treatment and steroid therapy [115]. These unwanted effects should be examined more carefully in order to better treat patients in these smaller categories considering that significant anti-tumor effects are observed and thus represent an exciting avenue of anti-glioma treatment.

#### Programmed death 1(PD1) and programmed death ligand 1(PD-L1)

PD-1 is a member of the CD28/CTLA-4 family, a type I membrane protein in the immunoglobulin superfamily, which has two ligands: PD-L1 and PD-L2. As PD-L1 is highly upregulated in macrophages and DCs, the interaction between PD-L1 and PD-1 on T cells negatively regulates T cell activation. PD-L1 has been shown to be mostly expressed on tumor cells, causing T cell apoptosis or anergy when it binds to PD-1 on T cells [106, 116].

The expression of PD-L1 is regulated mostly by the Akt pathway. Akt is activated through PI(3)K, which, in turn, upregulates the expression of PD-L1. This activation is negatively regulated by PTEN. So when a mutation or loss of PTEN (a common phenomenon of cancer cells) occurs, Akt is constitutively active, yielding high expression of PD-L1 [117].Through this mechanism, cancerous cells accumulate PD-L1 on their surface in order to protect them from the anti-tumor T cell response.

Anti PD-1/PD-L1 antibody-based therapies have seen significant success in clinical trials for different cancer treatments (Supplementary Table 1). A phase I clinical trial with PD-L1 antibody (BMS-936559/MDX-1105) demonstrated observable tumor regression in 6–17% of patients and prolonged disease stabilization in 12–41% patients at 24 weeks post-treatment in melanoma and kidney cancer therapies. In a PD-1 antibody-based clinical therapy for melanoma, non-small cell lung cancer, and renal cell cancer (BMS-936558/MDX-1106/nivolumab), 31%, 17%, and 29% of patients, respectively, showed marked therapeutic response [118]. A common side effect for this treatment involves fatigue albeit to the extent that it did not require suspending treatment and did not negate from the therapeutic efficacy [116]. In some patients, however, there were some serious side effects (pneumonitis and interstitial nephritis) that required adjunct treatment and the arrest of the anti-PD-1 treatment [118, 119] . Other than in this small collection of patients, the treatment was generally durable and, specifically in patients with PD-L1 positive tumor cell surface, an even better response was noted [119]. However, this approach, in recent phase III clinical trial did not show any therapeutic efficacy in patients with the recurrence of glioblastoma multiforme (GBM) and further analysis are needed (unpublished, data were presented at the World Federation of Neuro-Oncology Societies (WFNOS) meeting in 2017).

#### 3.2.3. 4-1BB and 4-1BBL

4-1BB (CD137) is an agonistic receptor, mostly present on activated T cells, natural killer cells (NKs), and antigen-activated regulatory T-cells [120]. This receptor belongs to the tumor necrosis factor (TNF) super family and binds to its ligand 4-1BBL (CD137L) present on APCs [121], such as B cells, DCs, and macrophages [122]. When 4-1BB binds to its ligand, 4-1BBL, it causes the production of costimulatory cytokines such as IFN-γ and IL-2, while also resulting in an increase in the cytotoxicity of the CD8 T-cells through T cell receptor (TCR) signaling [123]. For this reason, 4-1BB-based immunotherapy can elicit both innate and adaptive immune responses against cancerous cells, indicative of a high potency in a clinical setting.

Anti-4-1BB (anti-CD 137) antibody has been used in clinical trials as a single therapy as well as in combination with other check-point blockades or the typical standard of care, related to advanced solid tumors, as well as non-small cell lung cancer, colorectal cancer, head and neck cancer, multiple myeloma, and malignant melanoma. A phase I clinical study of anti-4-1BB (PF-05082566) showed a best overall response of stable disease in 22% (6/27) of patients with advanced cancers. The most common side effects that were reported with this treatment were fatigue, neutropenia, rash, and diarrhea. The most common clinical abnormalities were increased liver function (ALT and AST counts), leukopenia, thrombocytopenia, and hyperbilirubinemia [124].

#### Advantage and disadvantage of immunotherapeutic approaches in glioma clinical settings

As recognition of the importance of cancer immunotherapy has increased, many pre-clinical (animal models) and clinical investigations for both DC-based vaccines and immunomodulatory-based agents have been explored [23, 24, 27, 32, 108, 109, 124]. Given the rapid progress for this field, all immunotherapies except for anti-4-1BB (anti CD137) introduced in this review have been studied in clinical trials for glioma. Supplementary Table 1 lists the clinical trials for glioblastoma that have been completed or are currently in progress. However, there has been concern that these approaches could change the overall balance of the immune system in such a way that may lead to serious autoimmune side effects. Moreover, many agents of these currently developed check-point blockades cannot pass the BBB which could minimize the therapeutic efficacy for tumors of the CNS [3, 6, 7, 19, 24, 48, 52, 111, 115]. For that reason, it has been suggested that the local delivery of these agents via tumor-specific delivery vehicles around/in neoplastic cells may be immensely beneficial.

### Combinatory therapies: oncolytic adenoviral vehicle with local delivery of check point blockade based immunomodulator(s)

As mentioned in previous sections, both oncolytic virotherapy and immunotherapy have their advantages and disadvantages. Nevertheless, both approaches face the inherent hurdle that all glioma therapies face: a highly immunosuppressive microenvironment and guard by the BBB [23, 27, 108, 110]. However, combining these two therapeutic modules can maximize the eradication efficiency of glioma, since oncolytic adenoviruses can be customized to specifically replicate within and destroy tumor cells but also transiently (in significant yet controllable amounts) deliver therapeutic genes such as immunomodulator(s) that have been incorporated into the viral vectors.

Importantly, the accumulated evidence confirms the safety of oncolytic virus usage in clinical studies for the treatment of glioma [1, 6, 9, 11, 13, 19, 46]. As such, the oncolytic viruses and therapeutic immunomodulator(s) described previously can be investigated in conjunction and be expected to boost the efficacy of one another. For instance, therapeutic efficacy of Ad-Flt3L, oncolytic adenovirus expressing fms-like tyrosine kinase ligand (an immunostimulatory cytokine that recruits DCs to tumor sites) in combination with conditionally replicating oncolytic adenovirus (Ad-TK), and Ad-hIL12 have been shown to be more efficient in glioma animal models and are currently under investigation in a phase I clinical trial, Table 1. Both oncolytic adenovirus based therapy expressing IL-12 and 4-1BBL in a melanoma animal model system and oncolytic adenovirus expression of soluble PD-1 in a colon cancer model have demonstrated, albeit not in a glioma model system, highly enhanced combinatory therapeutic efficacy [121, 125].

**Figure 2:**
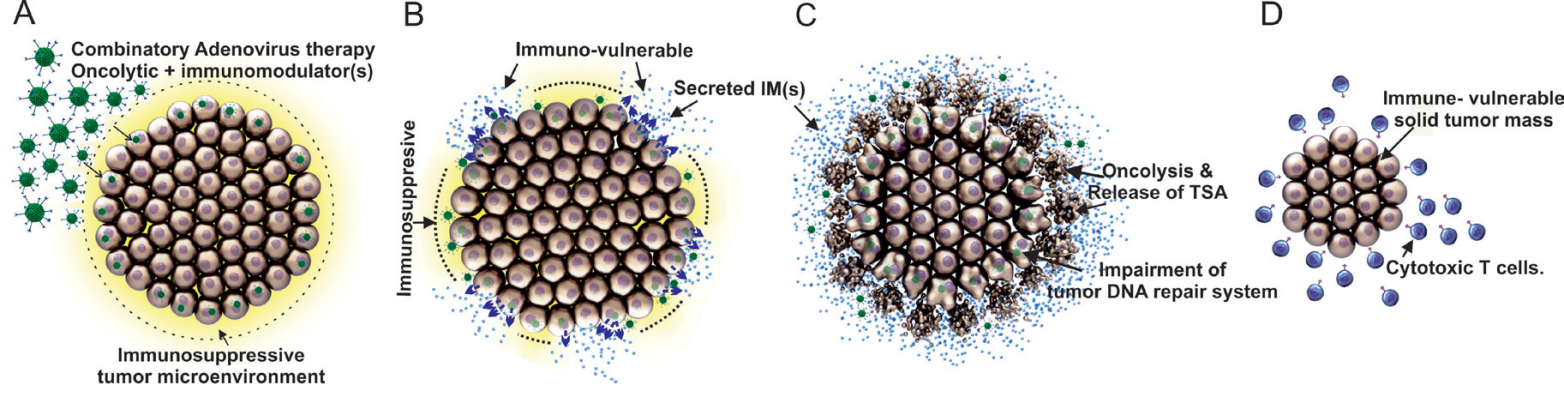
Mechanism of combinatory therapies for the treatment of glioma: oncolytic virotherapy paired with immunotherapy (**A**) Entry of oncolytic adenovirus into glioma cells (immunosuppressive microenvironment in yellow color). (**B**) Release of immunomodulator (IM) from oncolytic adenovirus infected glioma cells (a change of the tumor microenvironment from immunosuppressive to immunovulnerable via activation of cytotoxic T cells or removal of glioma cells’ immunosuppression capability affecting systemic immune repertoire). (**C**) Specific lysis of cancerous cells, induction of tumor specific antigens (TSA) release, and lastly, via the least known mechanism, impairment of tumor DNA repair system. (**D**) Eradication of the residual, immunovulnerable solid tumor mass by anti-cancer immune responses such as cytotoxic T cells.

Although immunological treatments are currently more pursued for clinical cancer therapies, the potential for even better cancer eradication can be achieved with the aid of oncolytic adenoviruses’ therapeutic capabilities. This new combinatorial platform can exploit multiple therapeutic features simultaneously. First, oncolytic viruses will enter cancerous cells and express immunomodulator(s) locally around the glioma. This will change the immunosuppressive microenvironment of glioma to an immuno-vulnerable environment by either enhancing the activation of cytotoxic T cells or removing the immunosuppressive protection of glioma cells. Second, oncolytic adenoviruses will replicate in a tumor-specific manner and lyse tumor cells in a coordinated manner. Third, oncolysis-mediated release of tumor antigen(s) will ensue for which the said antigen(s) will be engulfed by APCs such as DCs and processing/presentation will follow, eliciting an immune response. Fourth, although there are not many supporting mechanistic analyses, it has shown that oncolytic adenoviruses can also inhibit the DNA repair system in cancerous cells upon radiation therapy (radio-sensitization) [9, 10, 13, 95, 97]. It is therefore possible that oncolytic adenovirus-mediated impairment of DNA repair can cause the death of glioma cells in addition to the oncolytic activity [95, 97]. Lastly, after the loss of the immunosuppressive characterization of glioma via the first therapeutic effect, the un-penetrable, residual solid tumor mass can be eradicated completely by activated immune cells which are orchestrated by tumor-specific antigen presenting DCs, as shown in the Figure 2.

## CONCLUSIONS

This review outlines some of the challenges associated with traditional glioblastoma therapies like surgery and chemotherapy. These tumors are poorly circumscribed and invasive in nature, making traditional modes of treatment vastly less effective [9, 109]. Next, this paper describes some of the newly identified hurdles facing biologically active immunosuppressive agents and virotherapy for the treatment of glioma, such as the immunosuppressive tumor microenvironment and innate immunity to viral agents [6, 7, 9, 13, 22, 23, 32, 108, 109]. Nevertheless, there is encouraging data for immunotherapy and virotherapy in terms of their safety and/or effectiveness for other solid tumors. The benefit of combining these two therapeutic approaches to produce a synergistic anti-tumor effect and greater efficacy while maintaining patient safety is an attractive source of therapeutic consideration and thus warrants further investigation that can be applied for augmented therapeutic efficacy in anti-tumor effects and enhanced patient survival.

Adenoviral vectors have repeatedly been shown to have genomes that can be successfully modified for the generation of targeted, specific oncolytic virotherapy [6, 9, 11–13, 19]. Multiple steps in the viral infection and replication life cycle can be customized to create specific and selective agents for tumor destruction. For example, the cell surface receptor targeted by the viral fiber can be altered to enhance tumor infection, and the transcription of viral proteins can be restricted to cancerous cells, resulting in a viral construct with the ability to easily infect and selectively kill glioma cells [9–11, 13, 19, 46, 49]. However, limited distribution of the virus throughout the tumor and pre-existing systemic immunity and toxicity present hurdles for the efficacy of oncolytic virotherapy that must be overcome in order for the treatment to manifest into a therapy that has pronounced effectiveness [41, 42, 46, 49, 50].

Immunotherapy has been shown to be substantially effective for multiple solid tumor types. This approach is particularly enticing for glioma therapy given the strongly immunosuppressive nature of the tumor environment. There are multiple phases in the adaptive immune response that can be targeted: TA presentation with DCs, T cell checkpoint blockades to reduce the immunosuppression present in the tumor environment, and direct stimulation of activated T cells to name a few. Even these powerful therapies face challenges when utilized for glioma therapy like the restrictive nature of the BBB and systemic autoimmune generation [23, 25, 27, 32, 109, 110].

The ability for these seemingly divergent therapies, one being infection and the other an immune system activator, to complement each other may yield numerous benefits. For example, the autoimmune adverse effects of systemic checkpoint inhibition can be fatal, forcing some patients to discontinue immunomodulatory therapy [110, 111, 122]. But if these agents are only transiently delivered directly/locally to the tumor microenvironment, there should be a significantly reduced risk of systemic side effects. Although there is impairment of the BBB within the tumor, this impairment is not universally present throughout the entire tumor capillary bed. As such, systemic immunotherapies may not penetrate the entire tumor area. However, if the immunotherapeutic protein is actually produced when a viral vector that was directly injected into the tumor intra-operatively infects the tumor cells, themselves, then this is a barrier to therapy that can be overcome. Furthermore, it has been well established that viral cell death can generate an immune response and result in the exposure of tumor antigens to the immune system along with impairment of the DNA repair system in tumor cells such that combining this tumor-specific, immunologic cell death with immune stimulating therapy should be capable of producing a robust immune response [6, 9, 16, 40, 42, 46, 93].

There are still certain limitations that need to be addressed. Modifying the viral genome to carry the gene for a large immune system modulating protein may impair the viruses’ ability to replicate and spread. Additionally, turning the infected tumor cell into a “factory” for therapeutic immune-activating protein production requires that the cell is able to sufficiently generate enough therapeutic protein prior to its lysis. Recruiting the immune system to the tumor could potentially have the unwanted effect of limiting viral spread as well if the immune system begins to recognize and kill viral-infected cells. But if a robust immune response is generated against the tumor, there is potential for significant cerebral edema to occur, which can have catastrophic complications. However, these limitations can be also overcome by current and future pre-clinical and clinical research investigating more efficient and less toxic delivery for such treatment approaches in the treatment of glioblastoma.

The translational studies of oncolytic adenovirus-based therapies have made their way to many pre-clinical/clinical trials that have, by and large, shown pronounced safety and efficacy. Such approaches mechanistically induce a multi-level combination of tumor eradication activities: (1) specific lysis of cancerous cells, (2) induction of tumor specific antigen(s) release, and, (3) lastly, via the least known mechanism, impairment of tumor DNA repair system. However, based on current research in the field of oncolytic adenovirus-based therapy, even with well-crafted modifications to enhance affinity and tumor-restricted replication, there is still a need for additional support with other therapeutic approaches due to (1) the incapability to penetrate a solid mass of glioma and (2) inhibition of additional therapeutic immune activation/boost against tumor antigen(s) released via oncolytic activity by the immunosuppressive activity of glioma [6, 9, 13, 19, 95, 97].

As a rapidly progressing field in cancer therapy, immunotherapy is one of the most expanding and well-funded research areas at this time [23, 109]. DC-based vaccination and the use of immunomodulators (T cell check point blockades) aim to manipulate the immune system by either boosting anti-tumor immunity or extinguishing the immunosuppressive tumor microenvironment [23, 32, 93, 109, 110]. Both of these approaches have shown clinical efficacy, albeit with side effects such as autoimmunity [23, 24, 109]. However, in the case of current *ex vivo* DC-based tumor vaccine approaches, these applications are highly labor intensive and only allowed to be performed in good manufacturing practice (GMP) facilities [109, 111]. For future, optimal use of this application in a more logistically feasible manner, it is essential to generate single-step, *in vivo* DC-specific delivery vehicles that can facilitate highly efficient multi-epitope tumor antigen loading for maximal anti-tumor immunity. Additionally, problems resultant of the generation of systemic autoimmunity and the hindrance of delivery beyond the BBB are still faced. Specifically in immunomodulatory approaches, most immunomodulators in use cannot be transferred across the BBB and, in high concentration, have shown to be poorly tolerated by patients and are linked to the advent of autoimmunity as well as various other side effects [23, 109].

In light of this fact, the activation of the immune system and, specifically, local immune response activation are necessary to achieve better therapeutic effect in glioma treatment. Future endeavors to develop efficient and clinically relevant immunotherapies will require alleviation of these concerns in the context of both glioma abrogation and patient safety. Therefore, multi-level therapy such as combination of immunomodulators and viral vector approaches should be considered as a subject of further research investigations. In this approach, the infection of neoplastic cells with oncolytic viruses carrying immunomodulator(s), in addition to operating in an oncolytic capacity, introduces the immunomodulatory(s) to become locally expressed by the cancerous cells, thus allowing for a change of the tumor microenvironment from immunosuppressive to immune-vulnerable via activation of cytotoxic T cells or removal of glioma cells’ immunosuppressive capability affecting the systemic immune repertoire, followed by the oncolytic activity-mediated tumor eradication processes.

In conjunction with immunomodulatory approaches, it is expected that these therapies can boost the efficacy of one another. Future studies will be necessary to evaluate the effects of this combination in the hope that their conjunction will result in more pronounced anti-glioma effects that are pertinent to patient safety at the same time. Therefore, in the heart of this review, we propose the marriage of two sets of demonstrably efficacious and well-studied anti-tumor therapies for the treatment of glioma: oncolytic virotherapy paired with immunotherapy.

## SUPPLEMENTARY MATERIALS TABLE




