# Supplementary material for: Hitting the nail on the head: combining oncolytic adenovirus-mediated virotherapy and immunomodulation for the treatment of glioma
Source: Oncotarget. 2017 Sep 11;8(51):89391–405. doi: 10.18632/oncotarget.20810 (PMC5687697; doi:10.18632/oncotarget.20810)
Supplement: Supplementary file 1 [file oncotarget-08-89391-s001.pdf]

Hitting the nail on the head: combining oncolytic adenovirus-mediated virotherapy and immunomodulation for the treatment of glioma

SUPPLEMENTARY MATERIALS

Supplementary Table 1: Immunotherapy clinical trials in glioma. See_Supplementary_Table 1
