# Supplementary material for: Hitting the nail on the head: combining oncolytic adenovirus-mediated virotherapy and immunomodulation for the treatment of glioma
Source: Oncotarget. 2017 Sep 11;8(51):89391–405. doi: 10.18632/oncotarget.20810 (PMC5687697; doi:10.18632/oncotarget.20810)
Supplement: Supplementary file 2 [file oncotarget-08-89391-s002.docx]

**Supplementary Table 1: Immunotherapy clinical trials in glioma**

| **Trial number** | **Type of treatment** | **Phase** | **Therapeutic agent** | **Additional Information** |
| --- | --- | --- | --- | --- |
| NCT01808820 | DC vaccine | I | DC vaccine with tumor lysate and imiquimod | * In the process of recruiting participants |
| NCT02575261 | CAR | I/II | CAR-T cell immunotherapy for EphA2 Positive Malignant Glioma | * In the process of recruiting participants |
| NCT00612001 | DC vaccine | I | Autologous DC vaccine pulsed with glioma associated antigen peptide (GAA-DC). | * Completed; Median survival of patients on the GAA-DC trial was 14.5 months. In this study they also show that this this treatment was safe and without any dose-limiting toxicity. |
| NCT01204684 | DC vaccine | II | DC vaccine with tumor lysate and 0.2% resiquimod and adjuvant polyICLC | * Ongoing trial |
| NCT00766753 | DC vaccine | I /II | DC vaccine and second DC vaccine booster | * Ongoing trial |
| NCT01792505 | DC vaccine | I | DC vaccine and imiquimod cream | * Completed |
| NCT01144247 | CTL | I | Alloreactive CTL | * Completed: No results have been published. |
| NCT02049489 | DC vaccine | I | ICT-121 DC vaccine | * Ongoing trial |
| NCT02649582 | DC vaccine | I /II | DC vaccine with temozolomide chemotherapy | * In the process of recruiting participants |
| NCT02709616 | DC vaccine | I /II | personalized DC-based cellular vaccine | * In the process of recruiting participants |
| NCT02366728 | DC vaccine | II | Group1 unpulsed DC vaccine, group 2 CMV-specific DC vaccines with Tetanus-Diphtheria Toxoid (Td), and group 3 basiliximab infusions prior to the DC vaccine along with Td pre-conditioning | * In the process of recruiting participants |
| NCT00639639 | DC vaccine | I | DC vaccine with tetanus toxoid | * Ongoing trial |
| NCT02465268 | DC vaccine | II | DC vaccine with tetanus toxoid | * In the process of recruiting participants |
| NCT01082926 | T cell vaccine | I | Intratumoral Infusions of GRm13Z40-2, An Allogeneic CD8+ Cytolitic T-Cell Line Genetically Modified to Express the IL 13-Zetakine and HyTK | * Completed: No results have been published. |
| NCT00576446 | DC vaccine | I | DC vaccine with tumor lysate | * Completed: No results have been published. |
| NCT02772094 | DC vaccine | II | DC vaccine adjuvant immunotherapy | * Ongoing trial |
| NCT00068510 | DC vaccine | I | DC vaccine with tumor lysate | * Completed: Median overall survival with this treatment was 34.4 months. In this study they showed that this approach is safe and without any dose limiting toxicity. |
| NCT00846456 | DC vaccine | I /II | DC vaccine with mRNA from tumor stem cells | * Completed: Median overall survival in the treated group is 23.1 months and median overall survival of the control group was 7.86 months. They did not observe any significant treatment-related adverse effects with this treatment and the adverse effects that has been reported were in the normal range. |
| NCT00323115 | DC vaccine | II | DC vaccine pulsed with tumor lysate with temozolomide and radiotherapy | * Completed: Median overall survival with this treatment was 28 months. The only adverse effect observed was grade 2 unilateral neck pain after 1 cervical lymph node vaccine administration in one patient. |
| NCT00626483 | DC vaccine | I | RNA loaded DC vaccine with basiliximab | * Ongoing trial |
| NCT01280552 | DC vaccine | II | ICT-107 loaded DC vaccine | * Completed: Median overall survival with this treatment was 18.3 months and 16.7 months in the control DC group. This treatment was generally safe and well tolerated. |
| NCT00576641 | DC vaccine | I | DC vaccine pulsed with tumor peptide | * Completed: Median overall survival with this treatment was 38.4 months. DC vaccine that has been used in this study was non-toxic. |
| NCT02017717 | Anti CTLA4 | III | Nivolumab compared to Bevacizumab and nivolumab with or without Ipilimumab | * In the process of recruiting participants |
| NCT02311920 | Anti CTLA4 | I | Ipilimumab and/or nivolumab in combination with temozolomide | * In the process of recruiting participants |
| NCT01952769 | Anti PD1 | I /II | MDV9300 (Pidilizumab) | * Ongoing trial |
| NCT02529072 | Anti PD1 | I | Nivolumab monotherapy with or without DC vaccine therapy | * In the process of recruiting participants |
| NCT02658981 | Anti PD1 | I | Anti-LAG-3 Monoclonal Antibody BMS 986016 (urelumab) with or without nivolumab | * In the process of recruiting participants |
| NCT02423343 | Anti PD1 | I /II | Nivolumab with galunisertib | * In the process of recruiting participants |
| NCT02336165 | Anti PD1 | II | MEDI4736 | * In the process of recruiting participants |
| NCT02617589 | Anti PD1 | III | Nivolumab vs Temozolomide Each in Combination With Radiation Therapy | * In the process of recruiting participants |
| NCT02208362 | CAR | I | IL13RÎ±2-specific, hinge-optimized, 41BB-costimulatory CAR/truncated CD19-expressing Autologous T lymphocytes | * In the process of recruiting participants |
| NCT01454596 | CAR | I/II | Anti-EGFRvIII CAR transduced PBL with Aldesleukin, Fludarabine, and Cyclophosphamide | * In the process of recruiting participants |
| NCT02454634 | IDH1 vaccine | I | IDH1 peptide vaccine | * In the process of recruiting participants |
| NCT00045968 | DC vaccine | III | DCVaxÂ®-L | * Ongoing trial |
| NCT02010606 | DC vaccine | I | DC vaccine, in addition to standard temozolomide chemotherapy and involved field radiation therapy | * Ongoing trial |
| NCT00890032 | DC vaccine | I | DC vaccine loaded with BTSC | * Completed: No results have been published. |
| NCT00643097 | PEP-2 vaccine | II | PEP-3 vaccine with sargramostim and temozolomide | * Completed. Median progression free survival with this treatment: Arm I (ACTIVATE)14.2 months,  Arm II (ACT II STD) 12.1 months, and Arm III (ACT II DI) 11.6 months. This treatment was generally safe and well tolerated. |
| NCT02937844 | Anti PD1 | I | Anti-PD-L1 CSR T cells with cyclophosphamide and fludarabine | * In the process of recruiting participants |
| NCT02718443 | VEGFR-2 | I | VXM01 | * In the process of recruiting participants |
| NCT01522820 | DC vaccine | I | DC vaccine with DEC-205/NY-ESO-1 Fusion Protein CDX-1401 | * Completed: No results have been published. |
